# The role of predicted lean body mass and fat mass in non-alcoholic fatty liver disease in both sexes: Results from a secondary analysis of the NAGALA study

**DOI:** 10.3389/fnut.2023.1103665

**Published:** 2023-01-19

**Authors:** Maobin Kuang, Ruijuan Yang, Qiyang Xie, Nan Peng, Song Lu, Guobo Xie, Shuhua Zhang, Yang Zou

**Affiliations:** ^1^Department of Cardiology, Jiangxi Provincial People’s Hospital, Medical College of Nanchang University, Nanchang, Jiangxi, China; ^2^Jiangxi Cardiovascular Research Institute, Jiangxi Provincial People’s Hospital, The First Affiliated Hospital of Nanchang Medical College, Nanchang, Jiangxi, China; ^3^Department of Endocrinology, Jiangxi Provincial People’s Hospital, The First Affiliated Hospital of Nanchang Medical College, Nanchang, Jiangxi, China

**Keywords:** non-alcoholic fatty liver disease, predicted lean body mass, body mass index, predicted fat mass, LBM

## Abstract

**Objective:**

High body mass index (BMI) is an important risk factor for non-alcoholic fatty liver disease (NAFLD). However, the association of body composition such as fat mass (FM) and lean body mass (LBM) with NAFLD has not been adequately studied. The purpose of this study was to clarify the contribution of body composition FM and LBM to NAFLD.

**Methods:**

We analyzed data from 7,411 men and 6,840 women in the NAGALA cohort study. LBM and FM were estimated for all subjects using validated anthropometric prediction equations previously developed from the National Health and Nutrition Examination Survey (NHANES). Using multiple logistic regression and restricted cubic spline (RCS) to analyze the association and the dose-response curve of predicted LBM and FM with NAFLD in both sexes.

**Results:**

The prevalence of NAFLD in man and woman subjects was 27.37 and 6.99%, respectively. Predicted FM was positively and linearly associated with NAFLD in both sexes, with each 1 kg increase in predicted FM associated with a 27 and 40% increased risk of NAFLD in men and women, respectively. In contrast, predicted LBM was negatively associated with NAFLD in both sexes, with each 1 kg increase in predicted LBM reducing the risk of NAFLD by 4 and 19% in men and women, respectively. In addition, according to the RCS curve, the risk of NAFLD did not change in men when the predicted LBM was between 47 and 52 kg, and there seemed to be a saturation effect; further, the threshold value of the saturation effect was calculated to be about 52.08 kg by two-piecewise logistic regression, and the protective effect on NAFLD would be significantly enhanced when the man predicted LBM was greater than 52.08 kg.

**Conclusion:**

The current findings suggested that body composition LBM and FM had opposite associations with NAFLD in both sexes, with higher LBM associated with a lower risk of NAFLD and higher FM increasing the risk of NAFLD, especially in women.

## Introduction

Non-alcoholic fatty liver disease (NAFLD) is a clinicopathological syndrome characterized by excessive intrahepatocellular fat deposition due to etiologies other than alcohol and other well-defined factors of liver damage, and is an important risk factor for the development of end-stage liver disease, liver transplantation, and cardiovascular mortality (1, 2). The main causes of NAFLD are over nutrition and obesity (3), and epidemiological surveys have shown that the prevalence of NAFLD is increasing in parallel with obesity and diabetes (4); it is estimated that more than a quarter of the world’s population has NAFLD, and the prevalence is as high as 80% in obese people (5). However, NAFLD is not exclusive to the obese population, and a specific group of NAFLD has attracted increasing attention in recent years, namely non-obese/lean NAFLD (6); they have a normal or even low BMI, but this group of patients also has long-term intra- and extra-hepatic comorbidities and even a higher risk of liver-related events than obese NAFLD (7, 8).

High BMI is a recognized risk factor for NAFLD, however, the main risk factors and pathophysiological mechanisms of lean NAFLD are unknown and may be related to genetic factors and reduced skeletal muscle mass and function (9). Therefore, a key task is to further investigate the independent role of two major components of BMI, FM, and LBM, on the risk of NAFLD on the basis of clarifying the correlation between BMI and NAFLD risk. However, most similar studies have investigated the association of only one body composition with NAFLD risk (10–12), and only one cross-sectional study in a European elderly population investigated the independent effects of both FM and LBM on NAFLD risk (13). Considering the differences in body composition between different ethnic populations and the fact that there is currently no evidence of the correlation between body composition indicators (14) and NAFLD in the general population, the current study aimed to explore the independent association of LBM and FM with NAFLD in the general population in Asia based on the NAGALA study.

## Materials and methods

### Study design and population

The current study is a cross-sectional analysis of data from subjects in the NAGALA study cohort. The study design and purpose of the NAGALA cohort have been previously described in detail (15). In short, this research project has been continuously recruiting the general population who underwent health checkups at Murakami Memorial Hospital since 1994, and analyzing their examination data for the early detection of chronic diseases and their risk factors that have a significant impact on public health, and providing reference materials for the formulation of chronic disease prevention policies and clinical control. The NAGALA study has received ethical approval from the Murakami Memorial Hospital Ethics Committee and informed consent from the subjects (IRB2018-09-01), and the study dataset has been uploaded to the Dryad database by Prof. Okamura (16); other investigators were authorized to freely use the data from the study for secondary analysis without violating the terms of the database.

We extracted data from the Dryad database for 20,944 subjects recruited in the NAGALA cohort prior to 2016 and further excluded 1,131 subjects diagnosed with diabetes or fasting glucose above 6.1 mmol/L (impaired fasting glucose) at baseline, 416 subjects with liver disease (other than fatty liver), 1,952 subjects with excessive alcohol consumption (17), 2,321 subjects on medication at baseline, 863 subjects with incomplete examination data, and 10 subjects who withdrew from the study for unknown reasons according to the study objectives. The analysis of the data of all subjects in the current study complied with the Declaration of Helsinki, seeing STROBE statement (S1 Text), and was approved by the Ethics Committee of Jiangxi Provincial People’s Hospital (IRB2021-066).

### Collection and definition of anthropometric, clinical, and biochemical indicators

Information on age, lifestyle habits (smoking status, exercise habits, drinking status), sex, previous illnesses, and medication use were collected by professional medical staff based on a standardized questionnaire submitted by each subject, and standing systolic and diastolic blood pressure (S/DBP), waist circumference (WC), weight, height, and BMI were measured in the room using standard methods. In addition, lifestyle habits were stratified according to the following criteria: exercise habits: exercise at least once a week; smoking status: subjects were classified as none/past/present smokers according to their smoking history; and drinking status: no or small/light/moderate drinking according to weekly alcohol consumption (17).

Blood specimens from subjects in a fasting state (at least 8 h fasting) were analyzed using the automatic biochemical analyzer in the laboratory to obtain concentrations of various biochemical parameters, including fasting glucose (FPG), triglycerides (TG), gamma-glutamyl transferase (GGT), aspartate aminotransferase (AST), alanine aminotransferase (ALT), high-density lipoprotein cholesterol (HDL-C), Glycosylated hemoglobin (HbA1c), and total cholesterol (TC).

### Calculation of predicted FM and LBM

The predicted FM and LBM were calculated using anthropometric prediction equations (Table 1), which were developed and validated by Lee et al. from data extracted from the NHANES database of 14,065 subjects who had undergone dual-energy X-ray absorptiometry (DXA) examinations (18). Lee et al. incorporated the subject’s demographic information and anthropometric indicators into the multiple linear regression model as predictor variables, and continuously adjusted the included predictor variables to fit the linear regression models with the highest agreement with the actual FM and LBM measured by DXA. Ultimately, they found that linear regression models using height, WC, age, weight, and race as predictor variables had the highest consistency [LBM (women: R^2^ = 0.85; men: R^2^ = 0.91)] and [FM (women: R^2^ = 0.93; men: R^2^ = 0.90)].

**TABLE 1:** Anthropometric prediction equations for lean body mass (LBM) and fat mass (FM) developed from the National Health and Nutrition Examination Survey.

**Lean body mass**
**Men** 19.363 + 0.001 * age (year) + 0.064 * height (cm) + 0.756 * weight (kg)–0.366 * waist circumference (cm) –1.007
**Women** −10.683–0.039 * age (year) + 0.186 * height (cm) + 0.383 * weight (kg)–0.043 * waist circumference (cm)–0.340
**Fat mass**
**Men** −18.592−0.009 * age (year)−0.080 * height (cm) + 0.226 * weight (kg) + 0.387 * waist circumference (cm) + 1.050
**Women** 11.817 + 0.041 * age (year)–0.199 * height (cm) + 0.610 * weight (kg) + 0.044 * waist circumference (cm) + 0.325

### Diagnosis of NAFLD

As previously described (15), abdominal ultrasound was first performed on all subjects by a sonographer, and then a specialist gastroenterologist diagnosed NAFLD based on a combination of liver brightness, clarity of liver vessels, liver and kidney echo contrast and depth attenuation without any other information about the subjects (19).

### Statistical analysis

All analyzes in the current study were stratified by sex because of the sex-specific differences in body composition and the markedly different disease incidences and health outcomes associated with the sex (20). R language version 3.4.3 and Empower (R) version 2.0 were used for all analysis steps in this study and a two-sided *P* < 0.05 was considered significant.

Descriptive analysis: First, subjects of both sexes were divided into two groups according to whether they had NAFLD or not, and all data except lifestyle habits were described by mean (standard deviation) or median (25th, 75th percentile) according to whether they were normally distributed or not, while for lifestyle habits (smoking status, exercise habits, drinking status) data were described using frequency (%). Subsequently, to compare and quantify the differences between the Non-NAFLD and NAFLD groups, we calculated the weighted standardized difference values between the groups (>10% was considered significant) using the inverse probability of treatment weighting method (21).

Correlation analysis: First, all covariates were screened for collinearity using multiple linear regression analysis (22), and the final covariates with a variance inflation factor (VIF) greater than 5 were defined as collinear variables. Then, constructed four multivariate logistic regression models to examine the associations between predicted FM and LBM and BMI and NAFLD according to the recommendations of the STROBE guidelines (23), and in all models predicted FM and LBM were adjusted for each other and all collinear covariates were excluded. Model 1 was adjusted for age and lifestyle habits (smoking status, exercise habits, drinking status); model 2 considered the effect of liver function indicators (ALT, AST, GGT) on the association based on model 1; model 3 was further adjusted for glycemic parameters (FPG, HbA1c) based on model 2; finally, model 4 considered the effect of lipid parameters (TC, TG, HDL-C) on the association based on model 3.

Non-linear and threshold analyses: To further explore the effect of changes in predicted FM and LBM on NAFLD risk, this study fitted dose-response relationship curves between predicted FM and LBM and NAFLD risk based on model 4 using the RCS regression model with 4-knot. In addition, if a non-linear association was found between the predicted FM and LBM of both sexes and NAFLD, the two-piecewise logistic regression model was further used to find the optimal inflection point on the curve, i.e., the value of the inflection point corresponding to the model with the maximum likelihood estimate.

## Results

### Study subjects and characteristics

**FIGURE 1:**
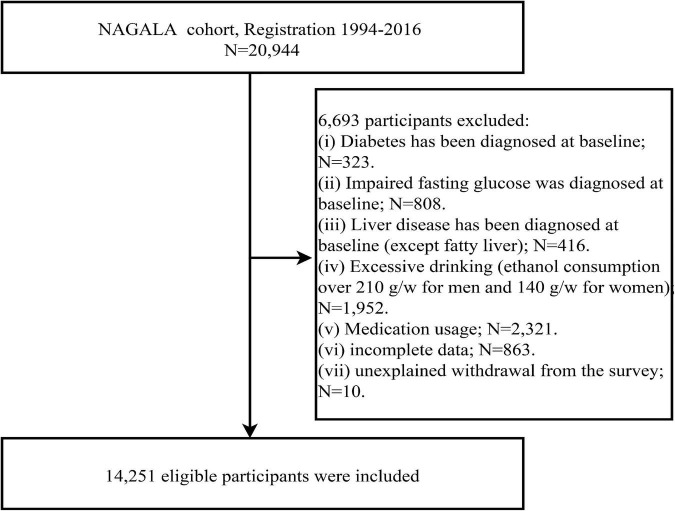
Flowchart of the selection process of study subjects.

After a further screening of the original data set, a total of 14,251 subjects were included in the current study (Figure 1), including 7,411 men with a mean age of 43.82 years and 6,840 women with a mean age of 43.22 years; the prevalence of NAFLD was 27.37 and 6.99% in men and women, respectively. Table 2 describes the basic data of subjects of both sexes grouped according to whether they had NAFLD or not. By looking at standardized difference values between the Non-NAFLD and NAFLD groups in both sexes, we found significant differences in most baseline parameters; subjects with NAFLD tended to have higher weight, BMI, predicted LBM, WC, predicted FM, ALT, GGT, AST, TG, TC, FPG, SBP, DBP, HbA1c levels, and lower HDL-C levels and less drinker, with obesity-related indicators predicted FM (standardized difference: 153% for women; 122% for men), WC (standardized difference: 154% for women; 122% for men), and BMI (standardized difference: 158% for women; 123% for men) having the largest standardized difference values. In addition, exercise habits differed significantly only between the man subject groups, age and height differed significantly only between the woman subject groups, and smoking status did not differ significantly between the Non-NAFLD and NAFLD groups in either sex. It is worth mentioning that the prevalence of NAFLD was much higher in men than in women, almost four times.

**TABLE 2:** Baseline characteristics of subjects grouped by sex and non-alcoholic fatty liver disease (NAFLD).

Characteristic	Men			Women		
	Non-NAFLD	NAFLD	Standardize diff. % (95% CI)	Non-NAFLD	NAFLD	Standardize diff. % (95% CI)
No. of subjects	5,382	2,029		6,362	478	
Age, year	42.00 (36.00–50.00)	43.00 (38.00–50.00)	5 (0, 10)	42.00 (36.00–49.00)	49.00 (41.00–54.00)	56 (46, 65)
Weight, kg	64.65 (8.34)	74.30 (10.56)	101 (96, 107)	51.86 (7.06)	63.17 (9.97)	131 (121, 141)
Height, m	1.71 (0.06)	1.71 (0.06)	5 (1, 10)	1.58 (0.05)	1.57 (0.05)	25 (16, 34)
BMI, kg/m^2^	22.12 (2.42)	25.48 (3.02)	123 (117, 128)	20.67 (2.57)	25.58 (3.57)	158 (148, 168)
WC, cm	77.99 (6.77)	86.62 (7.37)	122 (116, 127)	70.80 (7.30)	83.27 (8.86)	154 (144, 163)
LBM^&^, kg	49.30 (46.43–52.66)	52.98 (49.59–57.54)	75 (70, 81)	33.40 (31.41–35.47)	36.62 (33.93–39.26)	91 (82, 101)
FM^&^, kg	13.07 (10.32–15.87)	18.15 (15.43–21.35)	122 (117, 128)	16.64 (14.16–19.52)	24.27 (21.06–28.11)	153 (143, 162)
ALT, U/L	18.00 (14.00–23.00)	29.00 (22.00–41.00)	93 (87, 98)	13.00 (11.00–17.00)	19.00 (15.00–26.00)	63 (54, 73)
AST, U/L	17.00 (14.00–21.00)	21.00 (17.00–26.00)	54 (49, 60)	16.00 (13.00–19.00)	18.00 (15.00–22.00)	35 (26, 44)
GGT, U/L	17.00 (14.00–24.00)	24.00 (18.00–35.00)	44 (38, 49)	12.00 (9.00–14.00)	15.00 (12.00–20.00)	51 (41, 60)
HDL-C, mmol/L	1.30 (1.10–1.54)	1.11 (0.96–1.28)	68 (63, 73)	1.63 (1.40–1.89)	1.33 (1.16–1.56)	79 (70, 89)
TC, mmol/L	5.06 (0.84)	5.41 (0.85)	42 (37, 47)	5.05 (0.86)	5.56 (0.92)	57 (47, 66)
TG, mmol/L	0.80 (0.58–1.16)	1.32 (0.91–1.86)	75 (70, 80)	0.54 (0.40–0.77)	1.02 (0.73–1.38)	96 (87, 106)
FPG, mmol/L	5.25 (0.37)	5.42 (0.35)	48 (43, 53)	4.96 (0.38)	5.27 (0.40)	79 (70, 88)
HbA1c, %	5.13 (0.31)	5.27 (0.33)	45 (40, 50)	5.17 (0.32)	5.42 (0.33)	78 (69, 87)
SBP, mmHg	116.04 (13.16)	124.04 (14.46)	58 (53, 63)	108.42 (13.77)	120.71 (16.04)	82 (73, 92)
DBP, mmHg	72.88 (9.32)	78.44 (10.08)	57 (52, 62)	67.00 (9.48)	75.11 (10.22)	82 (73, 92)
Exercise habits, *n* (%)			13 (8, 18)			5 (0, 14)
No	4,300 (79.90%)	1,720 (84.77%)		5,351 (84.11%)	410 (85.77%)	
Yes	1,082 (20.10%)	309 (15.23%)		1,011 (15.89%)	68 (14.23%)	
Drinking status, *n* (%)			25 (20, 30)			16 (6, 25)
Non/small	3,731 (69.32%)	1,623 (79.99%)		5,986 (94.09%)	465 (97.28%)	
Light	1,096 (20.36%)	273 (13.45%)		376 (5.91%)	13 (2.72%)	
Moderate	555 (10.31%)	133 (6.55%)				
Smoking status, *n* (%)			6 (1, 11)			4 (0, 14)
None	1,952 (36.27%)	758 (37.36%)		5,609 (88.16%)	427 (89.33%)	
Past	1,538 (28.58%)	615 (30.31%)		382 (6.00%)	24 (5.02%)	
Current	1,892 (35.15%)	656 (32.33%)		371 (5.83%)	27 (5.65%)	

Values were expressed as mean (standard deviation) or medians (quartile interval), or *n* (%).

BMI, body mass index; WC, waist circumference; LBM, lean body mass; FM, fat mass; ALT, alanine aminotransferase; AST, aspartate aminotransferase; GGT, gamma-glutamyl transferase; HDL-C, high-density lipoprotein cholesterol; TC, total cholesterol; TG, triglyceride; FPG, fasting plasma glucose; HbA1c, glycosylated hemoglobin; SBP, systolic blood pressure; DBP, diastolic blood pressure.

^&^Derived from validated anthropometric prediction equations.

### Association of body composition and BMI with NAFLD

Supplementary Table 1 shows the results of collinearity screening, where WC, height, weight, and DBP were defined as collinear variables and excluded from the multivariate logistic regression models. To exclude the influence of confounding factors on the association as much as possible, we developed four stepwise adjusted multiple logistic regression models based on the epidemiology of NAFLD (Table 3). In model 1 with preliminary adjustment for age and lifestyle habits, predicted FM and BMI for both sexes were associated with increased risk of NAFLD, whereas predicted LBM was resistant to NAFLD risk for both sexes; in model 4, which further adjusted for liver function parameters, glycemic parameters, and lipid parameters, the direction of the associations between predicted FM and LBM and BMI and NAFLD remained the same and the magnitude of the associations changed only slightly, with each 1 kg increment in predicted LBM being associated with a 4% reduction in NAFLD risk in men (OR 0.96, 95% CI 0.94, 0.98) and a 19% reduction in NAFLD risk in women (HR 0.81, 95% CI 0.76, 0.87), whereas each 1 kg increment in predicted FM was associated with a 27% increased risk of NAFLD in men (HR 1.27, 95% CI 1.24, 1.31) and a 40% increased risk of NAFLD in women (HR 1.40, 95% CI 1.33, 1.47). Overall, body composition indicators predicted LBM and FM had opposite associations with NAFLD in both sexes, with higher predicted LBM associated with a lower risk of NAFLD, which was more protective in women than in men; in addition, higher predicted FM increased the risk of NAFLD, especially in women.

**TABLE 3:** The odds ratio of associations between predicted fat mass (FM) and lean body mass (LBM) and body mass index (BMI), and non-alcoholic fatty liver disease (NAFLD) risk.

	OR (95% confidence interval)			
	Model 1	Model 2	Model 3	Model 4
**Men**				
BMI	1.43 (1.40, 1.47)	1.32 (1.29, 1.36)	1.47 (1.43, 1.51)	1.41 (1.37, 1.45)
LBM^&^	0.92 (0.91, 0.94)	0.95 (0.93, 0.97)	0.95 (0.93, 0.97)	0.96 (0.94, 0.98)
FM^&^	1.43 (1.40, 1.47)	1.32 (1.29, 1.36)	1.32 (1.29, 1.35)	1.27 (1.24, 1.31)
**Women**				
BMI	1.58 (1.52, 1.63)	1.54 (1.48, 1.59)	1.48 (1.43, 1.54)	1.42 (1.37, 1.48)
LBM^&^	0.75 (0.70, 0.80)	0.77 (0.72, 0.82)	0.78 (0.73, 0.84)	0.81 (0.76, 0.87)
FM^&^	1.55 (1.48, 1.62)	1.51 (1.44, 1.58)	1.46 (1.39, 1.53)	1.40 (1.33, 1.47)

Model 1 adjusted for age, exercise habits, drinking status, and smoking status.

Model 2 adjusted for age, exercise habits, drinking status, smoking status, ALT, AST, and GGT.

Model 3 adjusted for age, exercise habits, drinking status, smoking status, ALT, AST, GGT, FPG, and HbA1c.

Model 4 adjusted for age, exercise habits, drinking status, smoking status, ALT, AST, GGT, FPG, HbA1c, TC, TG, and HDL-C.

Both predicted LBMI and predicted FMI were mutually adjusted for each other. Abbreviations as in Table 2.

^&^Derived from validated anthropometric prediction equations.

### Non-linear analysis and threshold effect analysis of predicted FM and LBM with NAFLD

**FIGURE 2:**
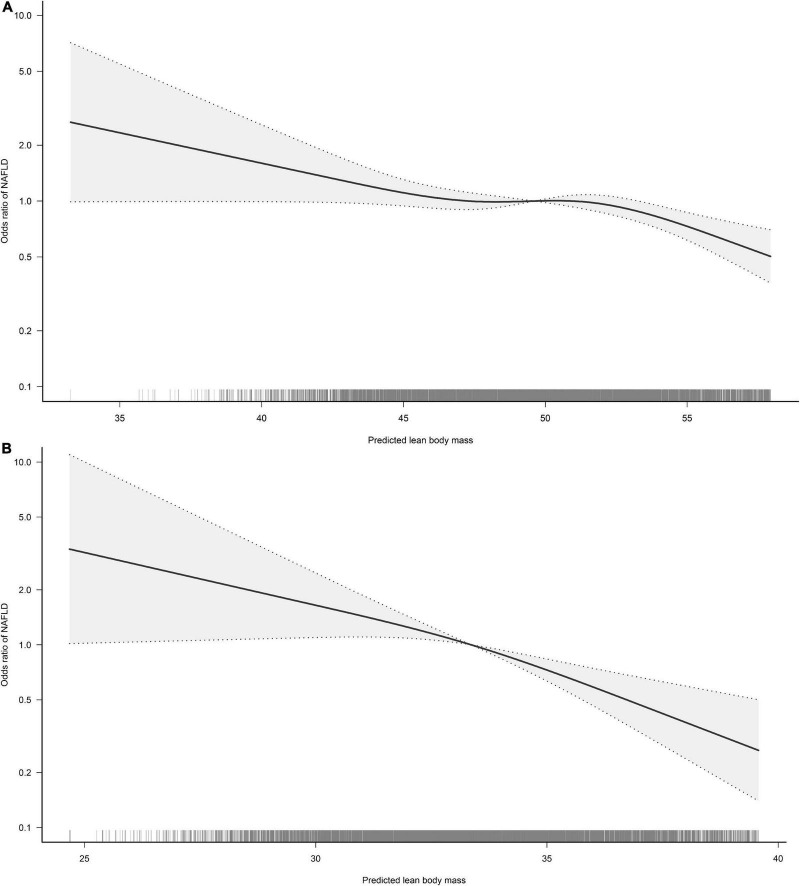
Restricted cubic spline analysis of predicted lean body mass (LBM) for the estimation of the risk of non-alcoholic fatty liver disease (NAFLD) in men **(A)** and women **(B)**. Adjusted for age, exercise habits, drinking status, smoking status, ALT, AST, GGT, FPG, HbA1c, TC, TG, HDL-C, and FM.

**FIGURE 3:**
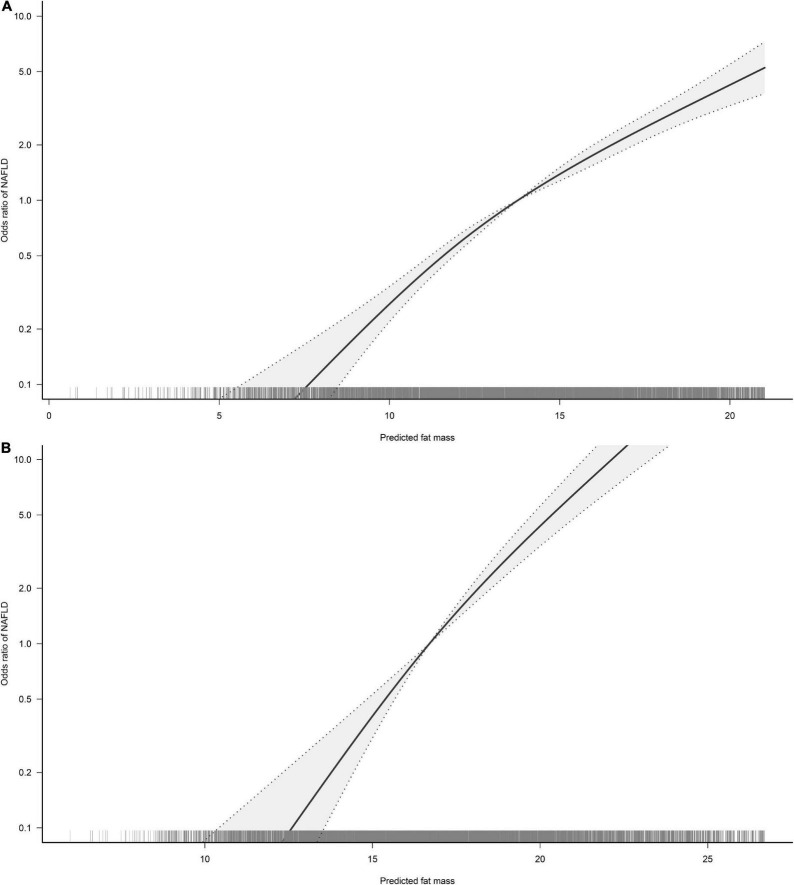
Restricted cubic spline analysis of predicted fat mass (FM) for the estimation of the risk of non-alcoholic fatty liver disease (NAFLD) in men **(A)** and women **(B)**. Adjusted for age, exercise habits, drinking status, smoking status, ALT, AST, GGT, FPG, HbA1c, TC, TG, HDL-C, and LBM.

To visualize the association between the continuous variables predicted FM and LBM and the risk of NAFLD, we nested the RCS regression model with 4-knot into model 4 to fit the dose-response curves between predicted FM and LBM and the risk of NAFLD in both sexes. A non-linear association of predicted LBM with NAFLD in men could be seen in Figure 2, where the risk of developing NAFLD did not change when the predicted LBM was between 47–52 kg, showing a saturation effect, while in women the predicted LBM was linearly associated with NAFLD. Moreover, from Figure 3, we observed that the predicted FM was linearly associated with the risk of NAFLD in both sexes. Subsequently, we further calculated the optimal inflection point on the dose-response relationship curve between predicted LBM and NAFLD risk in men using a two-piecewise logistic regression model by the point-taking method and found that when the predicted LBM was less than 52.08 kg, the OR value of each 1 kg increment associated with the risk of NAFLD in men was 0.98, while when the predicted LBM was greater than 52.08 kg, the protective effect on NAFLD was stronger, with an OR value of 0.94 (Table 4).

**TABLE 4:** Piecewise logistic regression examining thresholds for predicted lean body mass (LBM)-related non-alcoholic fatty liver disease (NAFLD) risk in men.

	NAFLD (OR, 95% CI)
	LBM^&^
**Fitting model by multivariate logistic regression**	
	0.96 (0.94, 0.98)
**Fitting model by two-piecewise logistic regression**	
The best inflection point	52.08
<inflection point	0.98 (0.95, 1.01)
>inflection point	0.94 (0.91, 0.97)

OR, odds ratios; CI, confidence interval; other abbreviations as in Table 2.

^&^Derived from validated anthropometric prediction equations.

## Discussion

In this large general population-based study, we analyzed the association of BMI and body composition indicators, predicted FM and LBM, with the risk of NAFLD. Consistent with the conventional view, this study found that BMI was associated with an increased risk of NAFLD in both sexes and that there was no significant difference in the effect of BMI on NAFLD in both sexes. However, this study revealed for the first time in the general population that predicted FM and LBM, components of BMI, were oppositely associated with NAFLD risk and had stronger effects on NAFLD in women than in men; where predicted LBM was negatively associated with the risk of NAFLD in both sexes and predicted FM was a common risk factor for NAFLD in both sexes. It is worth mentioning that the protective effect of predicted LBM on NAFLD in men was variable and will be further enhanced when the predicted LBM in men was greater than 52.08 kg.

In recent years, with the great increase in economic and material standards worldwide, a lifestyle of high energy intake and low energy consumption has become mainstream, and therefore obesity-related diseases have become the chronic diseases that have the greatest impact on the health of the general population, with almost parallel increases in the prevalence of diabetes, hypertension, metabolic syndrome, and NAFLD (4, 5, 24, 25). Previous studies have shown that insulin resistance (IR) is a core pathophysiological mechanism shared by NAFLD and these obesity-related diseases (3, 26, 27); IR is a pathological state in which the body develops compensatory hyperinsulinemia due to various factors that lead to reduced insulin-promoted glucose uptake and utilization (28). Therefore, further exploration of body parameters with important effects on insulin sensitivity may deepen our understanding of the relationship between obesity and NAFLD risk and provide new insights into the study of risk factors and pathogenic mechanisms of lean NAFLD.

Although a significant association between BMI, an indicator of obesity, and the risk of NAFLD has now been found in a large number of observational studies, BMI as a proxy measure of general obesity cannot explain the specific role of obesity on insulin sensitivity (29). Evidence from experimental studies suggested that the components of BMI, LBM, and FM, have different effects on insulin-induced regulation of body glucose (30–32). On the one hand, since LBM is overwhelmingly composed of skeletal muscle which is the main body tissue for insulin-induced glucose uptake, and the myofibers of skeletal muscle will release substances such as interleukins and irisin to maintain insulin sensitivity in skeletal muscle cells, a high LBM is more conducive to maintaining stable insulin-induced glucose metabolism (30, 31). On the other hand, excess FM will secrete excessive amounts of cytotoxic substances such as fatty acids, glycerol, and pro-inflammatory cytokines, which would increase IR in peripheral tissues (32); in addition, excessive ectopic deposition of adipose tissue in the liver and skeletal muscle has also been shown to cause IR in the liver and skeletal muscle (33). There is now a large body of evidence from observational studies showing that the two major components of BMI, FM, and LBM, are significantly and independently associated with the risk of obesity-related diseases such as diabetes, cardiovascular disease, and all-cause and cause-specific mortality (34–36), but the relationship between the two and NAFLD was only mentioned in a cross-sectional survey by Alferink et al. (13); their study found that FM and LBM were not significantly associated with NAFLD in an older male population in Europe, while in a normal weight older female population, LBM was significantly resistant to NAFLD risk (HR 0.84, 95% CI 0.75, 0.94), while FM was significantly associated with an increased risk of NAFLD (HR 1.16, 95% CI 1.03, 1.29). Considering the influence of ethnicity on body composition and that there is no evidence of the correlation between body composition indicators and NAFLD risk in the general population (14), the current study explored for the first time the association of body composition indicators predicted FM and LBM with NAFLD risk in a general population cohort from Asia.

The predicted FM and LBM in this study were calculated using anthropometric prediction equations, which have high predictive performance and have been used to calculate the LBM and FM of subjects in several large studies (18, 34, 36). The current study found significant sex differences in the effects of predicted FM and LBM on NAFLD in the general population, with each 1 kg increase in predicted FM increasing the risk of NAFLD by 40% in women and 27% in men, while each 1 kg increase in predicted LBM decreased the NAFLD risk by 19% in women and 4% in men. Sex differences in this correlation may be related to gender dimorphism in the effects of aging on NAFLD risk and different patterns of fat deposition due to differences in hormone levels in both sexes (37–39); From Table 2 we found that the age factor was balanced between the Non-NAFLD and NAFLD groups for men, while there was a significant difference between the two groups for women; the mean age of women in the Non-NAFLD group was 42 years, while the mean age of women in the NAFLD group was 49 years, which means that the shift in woman reproductive status that occurs with aging may have an additional impact on the risk of NAFLD. It is well known that women undergo dramatic changes in hormone levels before and after menopause, and that post-menopausal reduction in estrogen levels leads to lower levels of circulating IGF-1, DHEA, GH, and vitamin D as well as increased oxidative stress, and that all of these changes reduce skeletal muscle mass and function through the appropriate mechanisms (37, 40). Furthermore, high levels of estrogen in women can cause excess fat to be stored more in the subcutaneous tissues of the hips and thighs, a relatively healthy fat distribution, whereas men and post-menopausal women have lower levels of estrogen and excess fat tends to be deposited more in skeletal muscle tissue and abdominal visceral organs, dangerous fat distributions that pre-dispose to IR (38–41). Thus, in both men and post-menopausal women populations, except predicted FM, unhealthy fat distribution patterns also mediate a significant portion of BMI-related NAFLD risk.

It is worth mentioning that in the non-linear correlation analysis of this study we found a variable correlation between predicted LBM and NAFLD in men. When predicted LBM was less than 52.08 kg, each 1 kg increment in predicted LBM reduced the risk of NAFLD by 2% in men; after the predicted LBM increased to 52.08 kg, each 1 kg increment in predicted LBM was significantly and independently associated with a 6% reduction in the risk of NAFLD in men. In summary, given the relatively weak effect of body composition on the risk of NAFLD in the men population and the fact that general obesity indicator BMI remains a more important risk factor for NAFLD, we suggested that men should keep their LBM above 52.08 kg on the basis of diet control and weight loss to reduce the risk of NAFLD as much as possible. While the effects of LBM and FM on the risk of NAFLD were relatively greater in women, so performing appropriate resistance training to increase skeletal muscle mass while controlling the diet to reduce fat intake can effectively reduce the risk of NAFLD in women, and precise preventive interventions targeting the single body component may be a new strategy for NAFLD prevention in women.

### Study strengths and limitations

The greatest strength of this study is that it is the first to analyze the effect of body composition on the risk of NAFLD in a large sample of the general population, which will provide new insights into preventive interventions for NAFLD. In addition, this study also estimated the potential intervention threshold point of LBM for NAFLD prevention in men by non-linear correlation analysis and threshold effect analysis.

Of course, this study has some limitations: First, body composition indicators, predicted FM and LBM, were calculated by anthropometric prediction equations rather than the gold standard method DXA measurements; furthermore, although Lee et al.’s anthropometric prediction equations take into account the effect of race and have been used to calculate body composition in several published studies in Asian populations (42–44), the high predictive power of the prediction equations has not been directly confirmed in Asian populations at this time and needs to be validated in future studies. Second, the diagnosis of NAFLD was based on abdominal ultrasound images rather than the liver biopsy (19), however, it is unethical to perform an invasive test on the general population attending a health check-up. Third, since this study was a secondary analysis of previous research datasets, some risk factors for NAFLD, such as women’s reproductive status, cannot be further obtained, which may cause residual confounding; in addition, since the initial study did not perform bioelectrical impedance analysis on subjects to directly measure FM, this study could not compare the risk assessment ability for NAFLD of the fat mass index, an anthropometric measure with strong risk assessment power for NAFLD, with that of the predicted FM and LBM (45). Fourth, due to the cross-sectional study design, the causal association between body composition and NAFLD risk cannot be analyzed and needs to be verified in future large longitudinal cohort studies.

## Conclusion

In conclusion, the results of the current study suggested that increasing LBM can effectively reduce the risk of NAFLD in both sexes, especially in women, while men should keep their LBM above 52.08 kg to minimize the risk of NAFLD; moreover, excessive FM significantly increased the risk of NAFLD. Therefore, adding appropriate resistance training to increase skeletal muscle mass along with dietary control to reduce fat intake and weight loss is important to prevent NAFLD in both sexes.

## Data availability statement

The original contributions presented in this study are included in this article/Supplementary material, further inquiries can be directed to the corresponding authors.

## Ethics statement

The studies involving human participants were reviewed and approved by the Ethics Committee of Jiangxi Provincial People’s Hospital. Written informed consent for participation was not required for this study in accordance with the national legislation and the institutional requirements.

## Author contributions

YZ, SZ, MK, RY, and QX conceived the research, drafted the manuscript, and performed the statistical analysis. YZ revised the manuscript and designed the study. All authors read and approved the final manuscript.

## References

[1] Powell E Wong V Rinella M. Non-alcoholic fatty liver disease. *Lancet.* (2021) 397:2212–24. 10.1016/S0140-6736(20)32511-3 33894145

[2] Byrne C Targher G. NAFLD: a multisystem disease. *J Hepatol.* (2015) 62 (Suppl. 1):S47–64. 10.1016/j.jhep.2014.12.012 25920090

[3] Buzzetti E Pinzani M Tsochatzis E. The multiple-hit pathogenesis of non-alcoholic fatty liver disease (NAFLD). *Metabolism.* (2016) 65:1038–48. 10.1016/j.metabol.2015.12.012 26823198

[4] Younossi Z. Non-alcoholic fatty liver disease – a global public health perspective. *J Hepatol.* (2019) 70:531–44. 10.1016/j.jhep.2018.10.033 30414863

[5] Younossi Z Koenig A Abdelatif D Fazel Y Henry L Wymer M. Global epidemiology of nonalcoholic fatty liver disease-Meta-analytic assessment of prevalence, incidence, and outcomes. *Hepatology.* (2016) 64:73–84. 10.1002/hep.28431 26707365

[6] Ye Q Zou B Yeo Y Li J Huang D Wu Y Global prevalence, incidence, and outcomes of non-obese or lean non-alcoholic fatty liver disease: a systematic review and meta-analysis. *Lancet Gastroenterol Hepatol.* (2020) 5:739–52. 10.1016/S2468-1253(20)30077-7 32413340

[7] Golabi P Paik J Fukui N Locklear C de Avilla L Younossi Z. Patients with lean nonalcoholic fatty liver disease are metabolically abnormal and have a higher risk for mortality. *Clin Diabetes.* (2019) 37:65–72. 10.2337/cd18-0026 30705499 PMC6336127

[8] Hagström H Nasr P Ekstedt M Hammar U Stål P Hultcrantz R Risk for development of severe liver disease in lean patients with nonalcoholic fatty liver disease: a long-term follow-up study. *Hepatol Commun.* (2017) 2:48–57. 10.1002/hep4.1124 29404512 PMC5776871

[9] Wang A Dhaliwal J Mouzaki M. Lean non-alcoholic fatty liver disease. *Clin Nutr.* (2019) 38:975–81. 10.1016/j.clnu.2018.08.008 30466956

[10] Zhang Y Lu D Wang R Fu W Zhang S. Relationship between muscle mass/strength and hepatic fat content in post-menopausal women. *Medicina.* (2019) 55:629. 10.3390/medicina55100629 31554294 PMC6843176

[11] Lee J Lee H Lee B Kwon Y Lee J. Relationship between muscle mass and non-alcoholic fatty liver disease. *Biology.* (2021) 10:122. 10.3390/biology10020122 33562473 PMC7915258

[12] Hong H Hwang S Choi H Yoo H Seo J Kim S Relationship between sarcopenia and nonalcoholic fatty liver disease: the Korean sarcopenic obesity study. *Hepatology.* (2014) 59:1772–8. 10.1002/hep.26716 23996808

[13] Alferink L Trajanoska K Erler N Schoufour J de Knegt R Ikram M Nonalcoholic fatty liver disease in the Rotterdam study: about muscle mass, sarcopenia, fat mass, and fat distribution. *J Bone Miner Res.* (2019) 34:1254–63. 10.1002/jbmr.3713 31074909 PMC6852390

[14] Blue M Tinsley G Ryan ED Smith-Ryan A. Validity of body-composition methods across racial and ethnic populations. *Adv Nutr.* (2021) 12:1854–62. 10.1093/advances/nmab016 33684215 PMC8528114

[15] Okamura T Hashimoto Y Hamaguchi M Obora A Kojima T Fukui M. Ectopic fat obesity presents the greatest risk for incident type 2 diabetes: a population-based longitudinal study. *Int J Obes.* (2019) 43:139–48. 10.1038/s41366-018-0076-3 29717276

[16] Okamura T Hashimoto Y Hamaguchi M Ohobra A Kojima T Fukui M. *Data from: Ectopic fat Obesity Presents the Greatest Risk for Incident type 2 Diabetes: a Population-Based Longitudinal Study, Dryad, Dataset.* (2019). 10.5061/dryad.8q0p192 29717276

[17] Choi J Sohn W Cho Y. The effect of moderate alcohol drinking in nonalcoholic fatty liver disease. *Clin Mol Hepatol.* (2020) 26:662–9. 10.3350/cmh.2020.0163 32971586 PMC7641550

[18] Lee D Keum N Hu F Orav E Rimm E Sun Q Development and validation of anthropometric prediction equations for lean body mass, fat mass and percent fat in adults using the national health and nutrition examination survey (NHANES) 1999-2006. *Br J Nutr.* (2017) 118:858–66. 10.1017/S0007114517002665 29110742

[19] Hamaguchi M Kojima T Itoh Y Harano Y Fujii K Nakajima T The severity of ultrasonographic findings in nonalcoholic fatty liver disease reflects the metabolic syndrome and visceral fat accumulation. *Am J Gastroenterol.* (2007) 102:2708–15. 10.1111/j.1572-0241.2007.01526.x 17894848

[20] Hukportie D Li F Zhou R Zou M Wu X Wu X. Association of predicted lean body mass and fat mass with incident diabetic nephropathy in participants with type 2 diabetes mellitus: a post hoc analysis of ACCORD trial. *Front Endocrinol.* (2021) 12:719666. 10.3389/fendo.2021.719666 34777240 PMC8578879

[21] Sato T Matsuyama Y. Marginal structural models as a tool for standardization. *Epidemiology.* (2003) 14:680–6. 10.1097/01.EDE.0000081989.82616.7d 14569183

[22] Kim J. Multicollinearity and misleading statistical results. *Korean J Anesthesiol.* (2019) 72:558–69. 10.4097/kja.19087 31304696 PMC6900425

[23] Fitchett E Seale A Vergnano S Sharland M Heath P Saha S Strengthening the reporting of observational studies in epidemiology for newborn infection (STROBE-NI): an extension of the STROBE statement for neonatal infection research. *Lancet Infect Dis.* (2016) 16:e202–13. 10.1016/S1473-3099(16)30082-2 27633910

[24] Blüher M. Obesity: global epidemiology and pathogenesis. *Nat Rev Endocrinol.* (2019) 15:288–98. 10.1038/s41574-019-0176-8 30814686

[25] Lavie C Ozemek C Carbone S Katzmarzyk P Blair S. Sedentary behavior, exercise, and cardiovascular health. *Circ Res.* (2019) 124:799–815. 10.1161/CIRCRESAHA.118.312669 30817262

[26] Barber T Kyrou I Randeva H Weickert M. Mechanisms of insulin resistance at the crossroad of obesity with associated metabolic abnormalities and cognitive dysfunction. *Int J Mol Sci.* (2021) 22:546. 10.3390/ijms22020546 33430419 PMC7827338

[27] Jung U Choi M. Obesity and its metabolic complications: the role of adipokines and the relationship between obesity, inflammation, insulin resistance, dyslipidemia and nonalcoholic fatty liver disease. *Int J Mol Sci.* (2014) 15:6184–223. 10.3390/ijms15046184 24733068 PMC4013623

[28] Lebovitz H. Insulin resistance: definition and consequences. *Exp Clin Endocrinol Diabetes.* (2001) 109 (Suppl. 2):S135–48. 10.1055/s-2001-18576 11460565

[29] Sheng G Lu S Xie Q Peng N Kuang M Zou Y. The usefulness of obesity and lipid-related indices to predict the presence of non-alcoholic fatty liver disease. *Lipids Health Dis.* (2021) 20:134. 10.1186/s12944-021-01561-2 34629059 PMC8502416

[30] Katz L Glickman M Rapoport S Ferrannini E DeFronzo R. Splanchnic and peripheral disposal of oral glucose in man. *Diabetes.* (1983) 32:675–9. 10.2337/diab.32.7.675 6862113

[31] Pedersen B Febbraio M. Muscles, exercise and obesity: skeletal muscle as a secretory organ. *Nat Rev Endocrinol.* (2012) 8:457–65. 10.1038/nrendo.2012.49 22473333

[32] Kahn S Hull R Utzschneider K. Mechanisms linking obesity to insulin resistance and type 2 diabetes. *Nature.* (2006) 444:840–6. 10.1038/nature05482 17167471

[33] Seppälä-Lindroos A Vehkavaara S Häkkinen A Goto T Westerbacka J Sovijärvi A Fat accumulation in the liver is associated with defects in insulin suppression of glucose production and serum free fatty acids independent of obesity in normal men. *J Clin Endocrinol Metab.* (2002) 87:3023–8. 10.1210/jcem.87.7.8638 12107194

[34] Lee D Keum N Hu F Orav E Rimm E Willett W Comparison of the association of predicted fat mass, body mass index, and other obesity indicators with type 2 diabetes risk: two large prospective studies in US men and women. *Eur J Epidemiol.* (2018) 33:1113–23. 10.1007/s10654-018-0433-5 30117031

[35] Terada T Reed J Vidal-Almela S Mistura M Kamiya K Way K. Sex-specific associations of fat mass and muscle mass with cardiovascular disease risk factors in adults with type 2 diabetes living with overweight and obesity: secondary analysis of the look AHEAD trial. *Cardiovasc Diabetol.* (2022) 21:40. 10.1186/s12933-022-01468-x 35292039 PMC8925200

[36] Lee D Keum N Hu F Orav E Rimm E Willett W Predicted lean body mass, fat mass, and all cause and cause specific mortality in men: prospective US cohort study. *BMJ.* (2018) 362:k2575. 10.1136/bmj.k2575 29970408 PMC6028901

[37] Rolland Y Czerwinski S Abellan Van Kan G Morley J Cesari M Onder G Sarcopenia: its assessment, etiology, pathogenesis, consequences and future perspectives. *J Nutr Health Aging.* (2008) 12:433–50. 10.1007/BF02982704 18615225 PMC3988678

[38] Palmer B Clegg D. The sexual dimorphism of obesity. *Mol Cell Endocrinol.* (2015) 402:113–9. 10.1016/j.mce.2014.11.029 25578600 PMC4326001

[39] Tao Z Zheng L Smith C Luo J Robinson A Almeida F Estradiol signaling mediates gender difference in visceral adiposity via autophagy. *Cell Death Dis.* (2018) 9:309. 10.1038/s41419-018-0372-9 29472585 PMC5833393

[40] Maltais M Desroches J Dionne I. Changes in muscle mass and strength after menopause. *J Musculoskelet Neuronal Interact.* (2009) 9:186–97. 19949277

[41] Smith U. Abdominal obesity: a marker of ectopic fat accumulation. *J Clin Invest.* (2015) 125:1790–2. 10.1172/JCI81507 25932676 PMC4463217

[42] Liu L Ban C Jia S Chen X He S. Association of predicted fat mass, predicted lean mass and predicted percent fat with diabetes mellitus in Chinese population: a 15-year prospective cohort. *BMJ Open.* (2022) 12:e058162. 10.1136/bmjopen-2021-058162 35672066 PMC9174812

[43] Li M Lin J Liang S Huang S Wen Z Mo Z. Predicted fat mass, lean body mass, and risk of hypertension: results from a chinese male cohort study. *Obes Facts.* (2022) 15:638–47. 10.1159/000524653 35584613 PMC9669944

[44] Ge Y Liu J Zhang L Gao Y Wang B Wang X Association of lean body mass and fat mass with 1-year mortality among patients with heart failure. *Front Cardiovasc Med.* (2022) 9:824628. 10.3389/fcvm.2022.824628 35295256 PMC8918916

[45] Zhang S Wang L Yu M Guan W Yuan J. Fat mass index as a screening tool for the assessment of non-alcoholic fatty liver disease. *Sci Rep.* (2022) 12:20219. 10.1038/s41598-022-23729-1 PMC9684573 36418352

