# Supplementary material for: The role of predicted lean body mass and fat mass in non-alcoholic fatty liver disease in both sexes: Results from a secondary analysis of the NAGALA study
Source: Front Nutr. 2023 Jan 19;10:1103665. doi: 10.3389/fnut.2023.1103665 (PMC9894318; doi:10.3389/fnut.2023.1103665)
Supplement: Supplementary file 1 [file Table_1.DOCX]

Supplementary Table 1: Collinearity diagnostics steps.

|  | Variance inflation factor | | | | |
| --- | --- | --- | --- | --- | --- |
|  | Step 1 | Step 2 | Step 3 | Step 4 | Step 5 |
| LBM | 55344.2 | 116.8 | 5.1 | 3.7 | 3.7 |
| FM | 14615.8 | 32.8 | 6.5 | 5.3 | 5.3 |
| BMI | 124.7 | 90.9 | 8.9 | 8.7 | 8.7 |
| Age | 2.1 | 1.4 | 1.4 | 1.3 | 1.3 |
| WC | 6.5 | 6.4 | 6.3 | NA | NA |
| Weight | 80110.2 | NA | NA | NA | NA |
| Height | 84.3 | 47.1 | NA | NA | NA |
| Exercise habits | 1 | 1 | 1 | 1 | 1 |
| ALT | 4.2 | 4.1 | 4.1 | 4.1 | 4.1 |
| AST | 3.3 | 3.3 | 3.3 | 3.3 | 3.3 |
| GGT | 1.5 | 1.5 | 1.5 | 1.5 | 1.5 |
| HDL-C | 1.8 | 1.8 | 1.8 | 1.8 | 1.8 |
| TC | 1.5 | 1.5 | 1.5 | 1.5 | 1.5 |
| TG | 1.7 | 1.7 | 1.7 | 1.7 | 1.7 |
| FPG | 1.5 | 1.5 | 1.5 | 1.5 | 1.5 |
| HbA1c | 1.2 | 1.2 | 1.2 | 1.2 | 1.2 |
| SBP | 5.5 | 5.5 | 5.5 | 5.5 | 1.4 |
| DBP | 5.6 | 5.6 | 5.6 | 5.6 | NA |
| Drinking status | 1.2 | 1.2 | 1.2 | 1.2 | 1.2 |
| Smoking status | 1.4 | 1.4 | 1.4 | 1.4 | 1.4 |

Note-1: Variance inflation factor = 1/(1-R^2^). Abbreviations as in Table 2.

Note-2: The variables with Variance inflation factor >5 will be regarded as collinear variables and cannot be included in the multiple regression model.
